# Germline *TP53* mutations undergo copy number gain years prior to tumor diagnosis

**DOI:** 10.1038/s41467-022-35727-y

**Published:** 2023-01-05

**Authors:** Nicholas Light, Mehdi Layeghifard, Ayush Attery, Vallijah Subasri, Matthew Zatzman, Nathaniel D. Anderson, Rupal Hatkar, Sasha Blay, David Chen, Ana Novokmet, Fabio Fuligni, James Tran, Richard de Borja, Himanshi Agarwal, Larissa Waldman, Lisa M. Abegglen, Daniel Albertson, Jonathan L. Finlay, Jordan R. Hansford, Sam Behjati, Anita Villani, Moritz Gerstung, Ludmil B. Alexandrov, Gino R. Somers, Joshua D. Schiffman, Varda Rotter, David Malkin, Adam Shlien

**Affiliations:** 1 Genetics and Genome Biology, The Hospital for Sick Children, Toronto, Ontario Canada; 2 Institute of Medical Science, University of Toronto, Toronto, Ontario Canada; 3 Department of Molecular Cell Biology, The Weizmann Institute of Science, Rehovot, Israel; 4 Department of Medical Biophysics, University of Toronto, Toronto, Ontario Canada; 5 Vector Institute, Toronto, Ontario Canada; 6 Department of Laboratory Medicine and Pathobiology, University of Toronto, Toronto, Ontario Canada; 7 Division of Clinical and Metabolic Genetics, The Hospital for Sick Children, Toronto, Ontario Canada; 8 Department of Molecular Genetics, University of Toronto, Ontario, Canada; 9 Department of Pediatrics and Huntsman Cancer Institute, University of Utah, Salt Lake City, UT USA; 10 Peel Therapeutics, Inc., Salt Lake City, UT USA; 11 Department of Pathology, University of Utah School of Medicine, Salt Lake City, UT USA; 12 Departments of Pediatrics and Radiation Oncology, The Ohio State University College of Medicine, Columbus, OH USA; 13 Children’s Cancer Centre, Royal Children’s Hospital, Parkville, VIC Australia; 14 Murdoch Children’s Research Institute, Parkville, VIC Australia; 15 Department of Paediatrics, University of Melbourne, Melbourne, VIC Australia; 16 Michael Rice Cancer Centre, Women’s and Children’s Hospital, Adelaide, Australia; 17 South Australia Health and Medical Research Institute, Adelaide, Australia; 18 South Australian Immunogenomics Cancer Institute, University of Adelaide, Adelaide, Australia; 19 Wellcome Sanger Institute, Hinxton, UK; 20 Department of Paediatrics, University of Cambridge, Cambridge, UK; 21 Division of Hematology/Oncology, The Hospital for Sick Children, Toronto, Ontario Canada; 22 Department of Paediatrics, University of Toronto, Toronto, Ontario Canada; 23 European Molecular Biology Laboratory, European Bioinformatics Institute EMBL-EBI, Hinxton, UK; 24 Division of AI in Oncology, German Cancer Research Centre DKFZ, Heidelberg, Germany; 25 Department of Cellular and Molecular Medicine, Department of Bioengineering and Moores Cancer Center, University of California, San Diego, La Jolla, CA USA; 26 Department of Paediatric Laboratory Medicine, The Hospital for Sick Children, Toronto, Ontario Canada

**Keywords:** Cancer genetics, Cancer genomics, Paediatric cancer, DNA sequencing, Genetic predisposition to disease

## Abstract

Li-Fraumeni syndrome (LFS) is a hereditary cancer predisposition syndrome associated with germline *TP53* pathogenic variants. Here, we perform whole-genome sequence (WGS) analysis of tumors from 22 patients with *TP53* germline pathogenic variants. We observe somatic mutations affecting Wnt, PI3K/AKT signaling, epigenetic modifiers and homologous recombination genes as well as mutational signatures associated with prior chemotherapy. We identify near-ubiquitous early loss of heterozygosity of *TP53*, with gain of the mutant allele. This occurs earlier in these tumors compared to tumors with somatic *TP53* mutations, suggesting the timing of this mark may distinguish germline from somatic *TP53* mutations. Phylogenetic trees of tumor evolution, reconstructed from bulk and multi-region WGS, reveal that LFS tumors exhibit comparatively limited heterogeneity. Overall, our study delineates early copy number gains of mutant *TP53* as a characteristic mutational process in LFS tumorigenesis, likely arising years prior to tumor diagnosis.

## Abstract (web summary)

Li-Fraumeni syndrome (LFS) is associated with pathogenic germline *TP53* variants and predisposes patients to cancer; understanding the evolution and drivers of LFS-related tumours remains crucial. Here, the authors analyse 22 LFS tumours using whole-genome sequencing and reconstruct the evolution and timing of somatic driver alterations.

## Introduction

Li-Fraumeni syndrome (LFS) is an autosomal dominant cancer predisposition syndrome characterized by the early onset of a spectrum of childhood and adulthood cancers including adrenocortical carcinomas, soft tissue sarcomas, osteosarcomas, brain tumors and pre-menopausal breast cancers^1,2^. More than 70–80% of patients who fit the clinical definitions of LFS harbor pathogenic germline variants (mutations) in *TP53*, a tumor suppressor gene which coordinates the cellular response to DNA damage^3–6^. Current LFS patient management focuses on identifying and treating tumors at early stages through intensive multimodal surveillance protocols^7^. It is thought that *TP53* mutation carriers may be at a greater risk of developing radiation and chemotherapy-induced second primaries. However, there is little direct evidence to confirm this and while caution is recommended in the use of radiation in LFS patients, current treatment protocols for LFS-associated tumors do not differ from that of their sporadic counterparts. Several studies have shown an elevated number of structural variants in tumors from LFS patients, as well as frequent chromothripsis, and an enrichment for single base substitution (SBS) mutational signatures SBS3, SBS8 and SBS13^8–10^. Critical questions remain unanswered. What are the recurrent second hits following germline mutation of *TP53*? How do LFS tumors evolve? How do the effects of treatment manifest in subsequent primary tumors in these patients?

In this study, we perform whole-genome sequence (WGS) analysis on 22 tumors, across a range of LFS-spectrum tumor-types, from pediatric and young adult germline *TP53* mutation carriers. We observe frequent mutation of genes involved in Wnt signaling, PI3K/AKT signaling, epigenetic modifiers and homologous recombination (HR). Mutational signatures consistent with HR-deficiency are found in 4/4 tumors with somatic HR pathway mutations. We identify frequent chromothripsis and almost ubiquitous loss of heterozygosity (LOH) of *TP53*, with a gain of the mutant allele, which we time using clock-like mutational signatures to have occurred years before tumor diagnosis, likely in utero or early infancy. We found that *TP53* LOH occurs much earlier in mutational time in tumors arising in patients with a germline *TP53* mutation vs tumors with a somatic *TP53* mutation. We confirm our finding that *TP53* LOH is an early ubiquitous process in Li-Fraumeni syndrome, using primary fibroblast cell lines generated from skin biopsies of *TP53* mutation carriers. These primary fibroblast cell lines spontaneously underwent LOH within months of cell culture, gaining additional copies of mutant *TP53*. High levels of mutant p53 specific expression are detected in individual cells as early as 12 passages. Using bulk and multi-region WGS (46 total tumor regions), we reconstruct phylogenetic trees of tumor evolution, observing tumors arising in patients with a germline *TP53* mutation to exhibit proportionally larger sets of early truncal mutations. Overall, our study delineates early *TP53* LOH copy number gains in association with a later set of cooperating clonal somatic driver mutations as being the predominant mechanism of tumor evolution in our cohort of germline *TP53* mutation carriers. Many of the somatic driver mutations are of clinical relevance and we present preliminary data from an ongoing prospective sequencing study suggesting precision-oncology may have particular clinical utility in the care of LFS patients.

## Results

### The genomic landscape of LFS-associated tumors

**Fig. 1:**
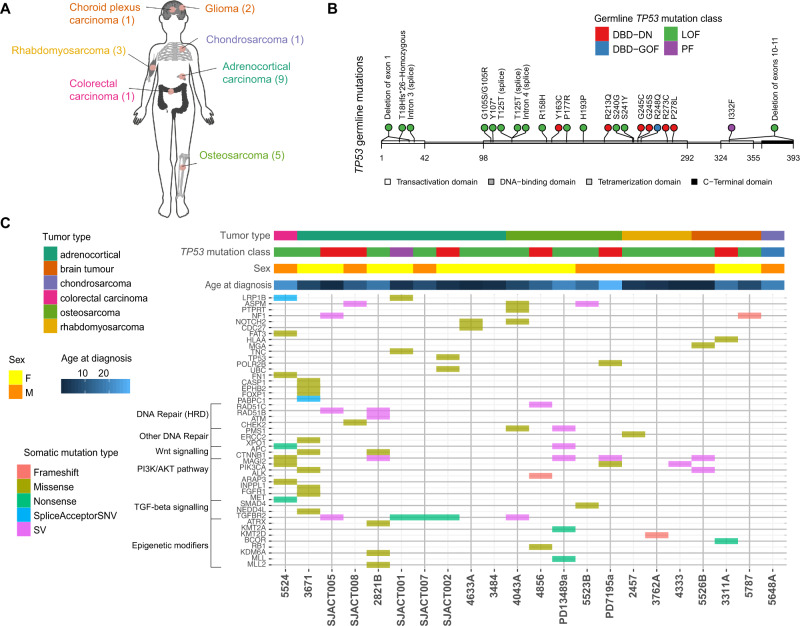
The mutational landscape of *TP53*-germline mutant childhood tumors. **A** *TP53*-germline mutant tumors included in study. (#) indicates number of tumors of each tumor type. **B** Location of germline mutations in *TP53* gene, colored by functional impact. DBD-DN DNA binding domain-dominant negative, DBD-GOF DNA binding domain- gain of function, LOF loss of function, PF partial function. **C** Top: Clinical covariates (tumor type, germline *TP53* mutation class, sex, age), Bottom: Predicted somatic driver mutations arranged by pathway and colored by type of mutation.

The primary genetic cause of Li-Fraumeni syndrome has been known for 30 years: germline mutations in *TP53* have been found in >70–80% of cases^11^. The somatic mutational events which cooperate with these germline *TP53* mutations, however, remain unclear. To address this, we performed somatic WGS analysis of 22 tumors (46 tumor regions) from childhood and young-adult patients [age 0–28, median age: 7.5] with confirmed pathogenic germline *TP53* mutations (Fig. 1A, B; Data S1). The samples analyzed spanned a range of LFS-spectrum tumor types (adrenocortical tumors [ACC], *n* = 9; osteosarcoma [OS], *n* = 5; rhabdomyosarcoma [RMS], *n* = 3; low grade gliomas [LGG], *n* = 2; choroid plexus carcinoma [CPC], *n* = 1; chondrosarcoma [CHS], *n* = 1; colorectal carcinoma [CRC], *n* = 1) (Fig. 1A; Data S1). Patients harbored a diverse set of germline *TP53* mutations. Twelve patients harbored heterozygous loss of function (LOF) mutations, 6 had heterozygous dominant negative mutations in the DNA binding domain (DBD-DN), one had a heterozygous partial function (PF) mutation and one had a heterozygous gain of function mutation in the DBD domain (DBD-GOF) (Fig. 1B). Notably, one patient (4333) presented with a homozygous *TP53* frameshift mutation (T18Hfs*26) related to consanguinity^12^, and a second (5526B) presented with two distinct *TP53* variants at the same base position in addition to a wildtype allele (G105S/G105R/WT), possibly due to a simultaneous mutation event during embryogenesis or aberrant clonal expansion^13,14^ (Fig. 1B, Fig. S1, Data S3, Data S4).

We began our analysis on the LFS tumor cohort by investigating somatic driver mutations occurring in addition to *TP53*. Analysis of predicted driver mutations including SNVs, indels and structural variants (SVs) revealed several recurrently mutated genes and pathways including *ATRX* (*n* = 4), *CTNNB1*/*APC* (*n* = 4), homologous recombination (*CHEK2, RAD51B, RAD51C*) (*n* = 4) and PI3K/AKT signaling (*PI3KCA, INPPL1*, etc.) (*n* = 8). (Fig. 1C). The *ATRX* mutations were predicted loss of function variants and these tumors displayed evidence of elongated telomeres, as determined by Telseq analysis comparing tumor vs matched normal blood. This represents an alternative lengthening of telomere phenotype, consistent with *ATRX* inactivation (Fig. S2)^15^.

### The mutational processes operating in LFS-associated cancers

**Fig. 2:**
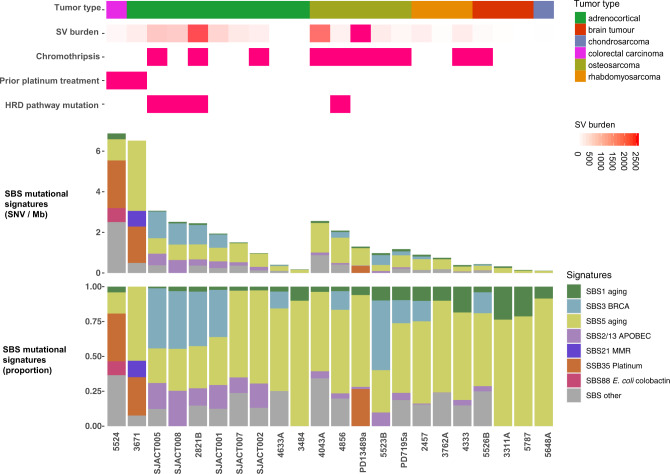
The mutational signatures of *TP53*-germline mutant childhood tumors. Top: Clinical and genomic features of the tumors (number of structural variants (SVs), presence of chromothripsis, prior platinum treatment, HRD pathway mutation). Bottom: SBS mutational signatures corresponding to COSMIC mutational signatures V3.2, scaled by mutation burden in tumor (above) and scaled to proportions of 1 for each tumor (below). X-axis depicts individual tumors. Source data are provided as a Source Data file.

To explore the processes causing accumulation of somatic mutations in LFS tumors, we performed mutational signature analysis using SigProfiler^16^. We identified 18 single nucleotide base substitution (SBS) signatures in the cohort. SBS1 (clock-like signature associated with spontaneous 5-methylcytosine deamination) and SBS5 (clock-like signature of unknown etiology) were present in all tumors (Fig. 2) and were found to correlate with patient age as has been shown previously in many adult tumor types (Fig. S3)^17^. SBS2 and 13 (APOBEC activity) were present in 16/22 tumors and were especially prevalent in ACCs. SBS21 (MMR-deficiency) was present in one tumor (3671), which was one of the two tumors with a mutation burden over 5 mut/Mb. SBS3 (homologous recombination-deficiency (HRD)/BRCA) was present in 10 tumors, including 5 of the 9 ACCs and all 4 of the tumors with mutations in an HR pathway gene. A high frequency of deletions at sites of microhomology, a hallmark of HRD, was also found in these tumors (Fig. S4). SBS18 (reactive oxygen species) was present in 9 tumors, although generally in low proportions. SBS88, a signature recently identified as being caused by the genotoxin, colibactin, produced by *pks*^*+*^ *E. coli*, was identified in the colorectal cancer, 5524^18^. SBS8, 16, 17a, 17b, 30, 38, and 41 (all of unknown etiology) were also present in one or more tumors. Interestingly, the two tumors with high mutation burdens (tumors: 3671 and 5524) exhibited large contributions of SBS35 (platinum therapy). Dinucleotide base substitution (DBS) analysis revealed that both tumors had large numbers of CT > AA/AC mutations in a pattern consistent with COSMIC dinucleotide base substitution DBS5 (Fig. S5). This pattern has previously been associated with platinum drug treatment of human cancer as well as in cell lines treated with carboplatin and cisplatin in vitro^19,20^. Interestingly, although sequenced samples from tumor 5524, a CRC, and 3671, an ACC, were obtained before any treatment had been initiated for these tumors, both patients had previously been diagnosed with separate primaries (OS and CPC, respectively) and had received cisplatin and/or carboplatin therapy >5 years before diagnosis of the later primary CRC and ACC. These results indicate that platinum-based chemotherapy treatment for LFS tumors can result in a substantial number of mutations detected in subsequent primary tumors and may directly contribute to their genesis.

### *TP53* mutant copy number gains, frequently associated with LOH, are an early and defining feature of LFS tumors

The most common recurrent somatic event observed across the cohort was LOH of the *TP53* locus, which occurred in 18/21 (86%) of the tumors that harbored heterozygous germline *TP53* mutations. Thus, the rate of *TP53* LOH in LFS tumors is substantially higher than previously reported^21^.This could be due to our focus on pediatric cancers and/or our use of the more sensitive method of WGS as compared to Sanger sequencing approaches used by others. Interestingly, the one sample with a *TP53* gain of function germline mutation (R248Q), was not observed to have undergone *TP53* LOH. Notably, in only one case, an ACC, was a somatic point mutation in *TP53* identified, suggesting that point mutations are not a common mechanism by which these tumors lose wildtype p53 function. Also, as previously reported in LFS tumors^10,22^, chromothripsis was prevalent in our cohort (10/22 cases) (Fig. 2).

**Fig. 3:**
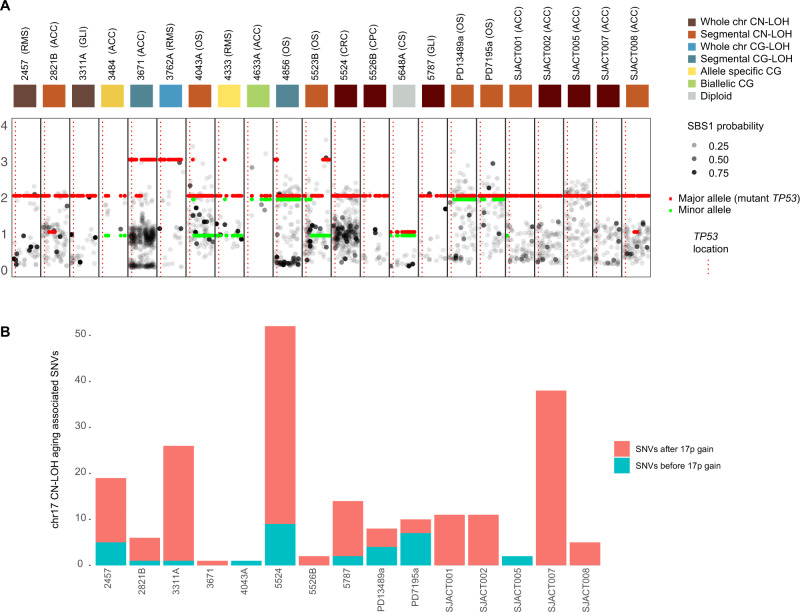
*TP53* LOH is an early event in the lifetime of LFS patients. **A** Copy number of somatic individual mutations (black and gray points) on chromosome 17 across cohort of *TP53*-germline mutant tumors. Each black box represents chromosome 17 for one tumor. X-axis for each black box is the position on chromosome 17; *Y*-axis shows the copy number. Red and green bars are major and minor copy number. Gray points are colored by probability of contributing to SBS1 (clock-like signature). Colored squares at top represent CN state of *TP53*. CG copy gain. CN copy number. **B** Number of aging associated mutations (SBS1 or SBS5) occurring before (blue bar) and after (red bar) *TP53* CN-LOH. X-axis depicts individual tumors. *Y*-axis depicts number of aging associated mutations. Source data are provided as a Source Data file.

Given the unexpectedly high rate of *TP53* LOH across our cohort, we sought to investigate this phenomenon in greater detail. To begin with, we classified *TP53* LOH according to the major and minor allele copy number as well as the size of the event (segmental, whole chromosome 17). LOH can occur with no change of copy number in the mutant allele of *TP53* (LOH), a single duplication of the mutant *TP53* allele (copy-neutral LOH), or multiple gains of the mutant *TP53* allele (copy-gain LOH). Our classification of *TP53* copy number revealed a diversity of states, ranging from whole chromosome copy-neutral LOH (*n* = 8), segmental copy-neutral LOH (*n* = 7), whole chromosome copy-gain LOH (*n* = 1), and segmental copy-gain LOH (*n* = 2) suggesting that *TP53* LOH occurs via an array of mechanisms in LFS patients (Fig. 3A). Surprisingly, none of the tumors exhibited LOH in the absence of a gain of the mutant allele of *TP53*. Of the three tumors in which we did not observe LOH, 2 had an allele-specific copy number gain of the mutant allele, with retention of the wildtype allele. Overall, 21/22 (95%) tumors exhibited a copy-number gain (2–3 copies) of a mutant *TP53* allele. This was supported in all cases by an increase in the variant allele fraction (VAF) of the *TP53* mutation in the tumor versus germline (Fig. S6). Of note, although complex chromothriptic events were common in our cohort, the *TP53* locus was not involved in these events, suggesting that secondary hits to *TP53* develop through other mechanisms.

The frequent gain of *TP53*-mutant copies presents an opportunity to time this event in the molecular evolution of the tumor. Clonal mutations occurring before duplication of the mutant chromosome are expected to have an average copy number of 2, whereas those occurring after the event should have an average copy number of 1 (or less if subclonal). Previous reports using this approach have indicated somatic copy number events may occur many years or even decades prior to tumor diagnosis^23^. To time *TP53* LOH we focused on germline *TP53* mutant tumors in which copy neutral LOH of the entire chromosome 17 had occurred (i.e. those in which we observed a loss of the wildtype *TP53*-containing chromosome and single copy gain of the chromosome harboring the mutant allele).

To estimate timing of LOH in these samples in the lifetime of the patient, we determined the mutation copy number of mutations on chromosome 17 (Fig. 3). We observed that in all cases, the majority of mutations had a copy number near 1, and were predicted to occur after duplication of the *TP53*-mutant copy of chromosome 17. Since SBS1 mutations accumulate as a clock-like signature over time, we sought to quantify the number of mutations along chromosome 17 contributing to SBS1 in tumors which had undergone CN-LOH. Specifically, we determined the probability for each mutation, being due to SBS1, based on its trinucleotide sequence (Fig. 3A). Strikingly, the vast majority of mutations due to the SBS1 for all tumors with CN-LOH of chromosome 17 occurred after duplication of the mutant containing chromosome. Thus, CN-LOH occurs many years before tumor diagnosis, likely *in utero* or early in life (Fig. 2B).

**Fig. 4:**
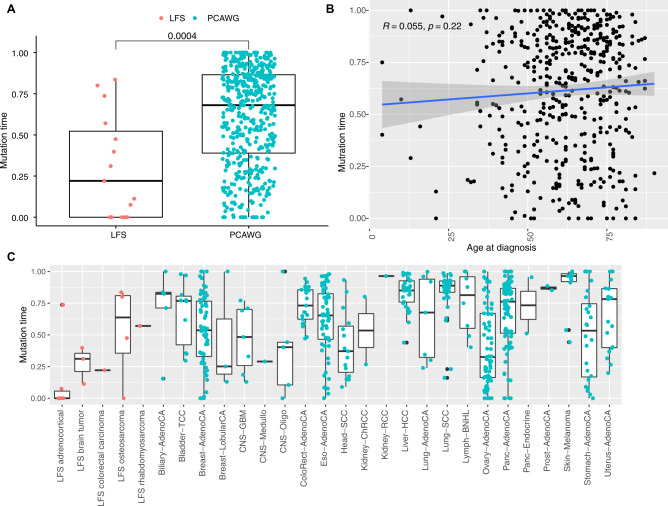
*TP53* LOH occurs earlier in mutational time in LFS tumors than in *TP53* somatic mutant tumors. **A** LFS tumors (left) and PCAWG *TP53* somatic mutant tumors (right) plotted by *TP53* LOH mutation time (*Y*-axis). Wilcoxon 2-tailed *p*-value shown. Boxplots show quartiles with whiskers representing 1.5x interquartile range. *N* = 501 biologically independent samples from distinct patients. **B** Age at diagnosis vs *TP53* LOH mutation time for PCAWG *TP53* somatic mutant tumors (*Y*-axis). Pearson correlation shown. Shaded area represents 95% CI. **C** *TP53* LOH mutation time by tumor type and LFS status. Red points are LFS tumors, blue points are somatically mutant tumors in PCAWG. Boxplots show quartiles with whiskers representing 1.5x interquartile range. Source data are provided as a Source Data file.

Having established that *TP53* LOH occurs early in these tumors, we next compared the timing of *TP53* LOH in our cohort to what occurs in spontaneous tumors in non-LFS individuals. We applied MutationTimeR, an algorithm that determines the fraction of mutations occurring before copy-gain events^24^, to the *TP53* CN-LOH events in our LFS cohort and all *TP53* CN-LOH events in the somatic *TP53* mutant PCAWG dataset. An example of MutationTimeR applied to one of the LFS tumor samples is shown in Supplementary Fig. 7. We found that the *TP53* LOH occurred earlier in mutational time in LFS tumors compared to that seen in somatic *TP53* mutant samples in PCAWG (Fig. 4A). In the LFS tumors, *TP53* LOH usually occurred in the first 25% of mutational time, whereas in the somatic *TP53* mutant tumors *TP53* LOH usually occurred in the second half of mutational time (Fig. 4A). There was no significant effect of age of tumor diagnosis on mutational timing of *TP53* LOH (Fig. 4B). Interestingly, the timing did differ by tumor type within the LFS cohort (Fig. 4C). The adrenocortical carcinomas had the earliest timing of *TP53* LOH, with almost no mutations occurring before this event. On the other hand, the osteosarcomas had the latest timing of LOH, with almost 50% of mutations occurring before LOH.

Our findings suggest that copy number gains of mutant *TP53* are an early and near-ubiquitous event in LFS patients’ tumors. Given this, we sought to determine if we could detect mutant *TP53* copy number gains and/or other mutational events not only in tumors, but also in non-malignant cells from LFS patients. To investigate this, we analyzed normal colonic tissue taken during the colorectal cancer surgery for tumor 5524, performing ultra-deep whole exome sequencing (5,517x mean depth) as well as ddPCR of all driver mutations identified in the tumor, in addition to two heterozygous SNPs at the TP53 locus. We were unable to identify driver mutations from the tumor in the nonmalignant tissue and the sensitivity of ddPCR was unable to resolve a significant shift in the allelic ratio (from 50:50) in the *TP53*-associated heterozygous SNPs (Fig. S8). This analysis was limited by our sensitivity to detect rare events in an analysis of a large number of non-clonal cells. To overcome this challenge and characterize the frequency and timing of spontaneous *TP53* LOH in LFS patients we leveraged the unique biology of LFS patient derived fibroblasts, which spontaneously immortalize in culture^25^. We passaged primary fibroblast cell lines obtained from skin biopsies of patients with germline R248Q *TP53* mutations. We then performed WGS at an average of 40X depth on early (12–14 passages) and late primary fibroblast passages (37–38 passages) as well as on matched blood-derived DNA. In 2 of the 3 patients’ fibroblasts analyzed, we identified copy number gains of the mutant allele of *TP53* by WGS in the late passage (Fig. S9). In the first cell line we observed multiple copy gains of the mutant allele of *TP53* in 17p as well as a single copy gain of 10q. In the second cell line we observed a large number of copy number changes across the genome with a genome duplication event resulting in 4 copies of the mutant allele of *TP53* and 2 copies of the wildtype allele. The third cell line showed a quiet diploid genome, with no evidence of copy number changes of *TP53* by WGS.

To more precisely determine LOH timing and whether *TP53* LOH was present in a subset of cells at earlier passages, but simply not detected by WGS, we performed immunofluorescence using a mutant p53 specific antibody at early, middle and late passages. Previous studies have shown that mutant p53 is stabilized and accumulates in the nucleus following *TP53* LOH^26^. We found that mutant p53 was expressed at high levels in individual cells as early as passage 12, subsequently becoming the dominant population of cells by passage 36 (Fig. S10).

Overall, these results confirm that copy number gains of mutant *TP53* occur spontaneously in LFS patient cells and can readily outcompete diploid clones in a small number of generations. These results align with our model in which *TP53* mutant copy number gain is an early spontaneous event in LFS tumorigenesis.

### The clonal evolution of LFS tumors is marked by a large clonal population of mutations

**Fig. 5:**
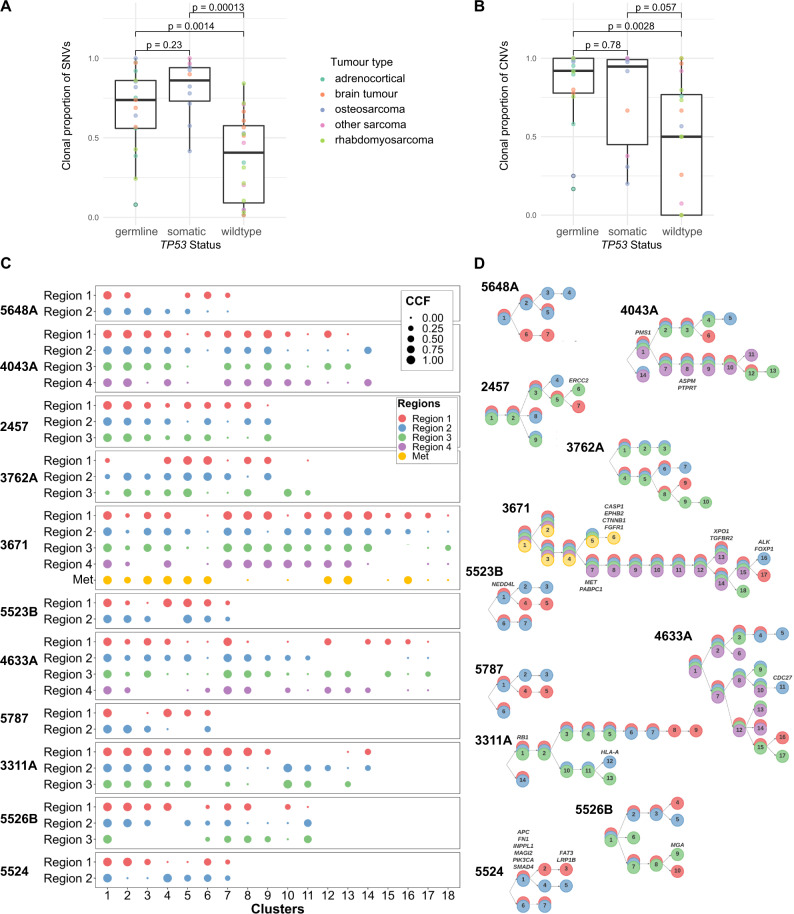
*TP53* mutant tumors have high proportion of clonal mutations. **A** Boxplots showing proportion of SNVs per tumor (colored circles), which are clonal. Wilcoxon 2-tailed *p*-value shown. Colors indicate tumor type. Boxplots show quartiles with whiskers representing 1.5x interquartile range. *N* = 47 biologically independent samples from distinct patients. *X*-axis depicts *TP53* status, *Y*-axis depicts proportion of SNVs which are clonal. **B** Boxplots showing proportion of CNVs per tumor (colored circles), which are clonal. Wilcoxon 2-tailed *p*-value shown. Boxplots show quartiles with whiskers representing 1.5x interquartile range. Colors indicate tumor type. -axis depicts *TP53* status, *Y*-axis depicts proportion of CNVs which are clonal. **C**, **D** Phylogenetic reconstructions of LFS tumors using multiregion WGS (**C**) Circle plots, with subclonal cluster number on *X*-axis and tumor region on *Y*-axis. Circles are colored by tumor region. Circle diameter corresponds to cancer cell fraction (CCF) for each cluster for each tumor region. **D** Phylogenetic tree reconstructions of each multiregion sequenced tumor with colors corresponding to tumor region, numbers corresponding to cluster number and annotated with location of driver mutations. Source data are provided as a Source Data file.

We next sought to investigate the role of *TP53* mutations in shaping childhood cancer evolution by examining the clonality of somatic mutations. To do this we analysed the *TP53* germline-mutant cohort alongside *TP53* somatic-mutant (*n* = 15) and *TP53*-wildtype (*n* = 33) tumor samples, as part of the SickKids Cancer Sequencing (KiCS) childhood precision medicine program^27^. These tumors were selected to match the LFS tumor cohort. Using PhyloWGS in single-sample mode we reconstructed the phylogenetic trees for all tumors. The proportion of clonal versus subclonal SNVs and CNVs differed markedly based on *TP53* mutation status (Fig. 5A). When comparing the proportion of SNVs and CNVs in the major clone to those which were subclonal, *TP53* germline-mutant and *TP53* somatic-mutant tumor samples exhibited a significantly higher proportion of mutations which were clonal as compared to *TP53* wildtype tumors (Fig. 5A, B).

To understand how the timing of mutations in LFS tumors was reflected spatially within tumors, we performed micro-dissection of frozen tumor tissue and applied WGS to multiple regions. Using PairTree we reconstructed the tumor evolution across these multiple tumor regions in LFS (Fig. 5C, D, Fig. S11) and non-LFS tumors (Fig. S12). Interestingly, while these tumors appeared to be highly clonal when analyzing single tumor regions, we found a larger degree of subclonality when assessing SNVs and indels with multiregion sequencing. Whereas SNVs and indels displayed intratumor heterogeneity, copy number events and SVs were highly clonal even across different tumor regions. Chromothriptic events were also confined to the truncal clone, with nearly identical copy number and structural variant patterns across all tumor regions observed in these tumors (Fig. S13). In addition to multiregion sequencing analysis of primary tumors, we also examined the clonal evolution of tumor 3671 through analysis of 4 primary adrenocortical carcinoma tumor regions as well as a lung metastasis. Interestingly we found 4 driver mutations specific to the metastasis (Fig. 5C, D) and copy number profile analysis showed moderate divergence of the lung metastasis from the 4 primary regions (Fig. S14).

## Discussion

**Fig. 6:**
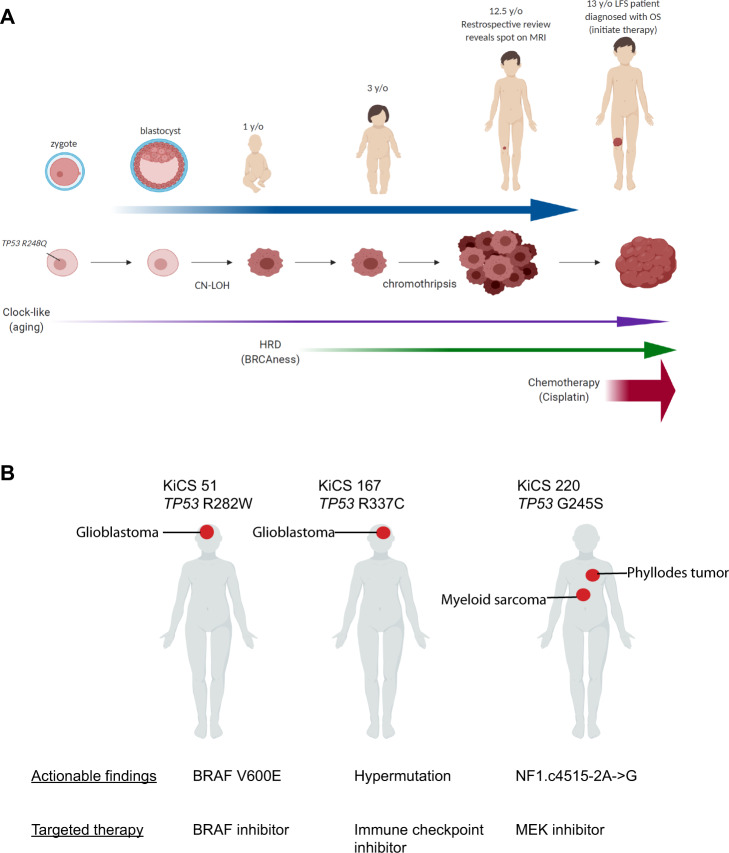
Translating our molecular understanding of LFS tumorigenesis into patient care. **A** Proposed model of the temporal occurrence of common events involved in the initiation and progression of tumorigenesis in patients with *TP53* germline mutations. Colored arrows indicate common mutational processes operating in premalignant and malignant cells and the approximate times in tumorigenesis these processes are active. **B** LFS patients enrolled in the KiCS precision medicine program and their actionable findings with corresponding potential targeted therapies.

In this study, we present the mutational landscape of tumors arising in *TP53* mutation carriers and delineate the somatic mutational processes and patterns of clonal evolution (Fig. 6A). We observed a common set of early clonal mutational mechanisms including frequent chromothripsis, and near ubiquitous early gain of mutant *TP53*. The early copy number gain associated *TP53* LOH in LFS tumors relative to somatic *TP53* mutant cancers may be explained by the increased selective pressures for a “second hit” to *TP53* when a first hit exists from conception. Timing *TP53* mutant copy gains may be a potential method for helping to determine pathogenicity of *TP53* variants of unknown significance. Phylogenetic reconstruction of LFS tumors revealed an enrichment for a high proportion of clonal truncal mutations in germline *TP53* mutant childhood cancers. Further studies will be needed to determine whether adult LFS cancers, especially breast cancers, bear the same genomic hallmarks of germline *TP53* mutations which we find in this cohort, as well as to assess the clinical utility of precision oncology in the context of LFS. Especially important will be the evaluation of additional tumors from LFS patients who have undergone treatment for earlier tumors to assess mutational signatures associated with earlier treatments. Indeed, the identification of a cisplatin mutational signature arising from treatment of an earlier tumor, raises the possibility of in vitro studies to investigate the mutational signatures induced by various therapies on LFS-patient derived cell lines. These may be useful in delineating if and how treatment modalities should be adjusted for LFS patients to avoid additional tumors, a common question among physicians and LFS patients alike.

Given the often poor outcomes of tumors in LFS patients using conventional treatments, we reason that these patients could be candidates for targeted therapy. In fact, in our KiCS prospective cohort study of ‘hard-to-treat’ or relapsed childhood cancer we identified 3 patients with pathogenic *TP53* germline mutations (Fig. 6B). All three patients underwent a clinically validated matched germline/somatic cancer panel analysis of >800 genes. Notably, clinically actionable results were found for all three patients, including a *BRAF* V600E mutation, which would make the patient a candidate for a BRAF inhibitor, hypermutation which would suggest the utility of immune checkpoint blockade and an *NF1* mutation, which would make the patient a candidate for a MEK inhibitor.

In our investigation, we found chemotherapy treatment of LFS tumors results in mutations detected in subsequent primary tumors. While not definitive proof of their causal role in tumorigenesis, the extent to which subsequent primary tumors are caused by previous chemotherapy (especially platinum-based) and radiotherapy in LFS patients merits further study. Our finding of a *pks* + *E coli* genotoxin signature in an LFS patient’s colorectal cancer is intriguing and suggests an opportunity for future studies to explore this as a mechanism by which LFS patients may develop such tumors. Understanding to what degree such bacteria pose an additional risk of gastrointestinal malignancies in LFS patients may be of value in designing cancer prevention strategies in this population. Our results suggest that LFS patients tumors frequently harbor targetable driver mutations, which may be ideal candidates for targeted treatments, and may be less likely to result in additional mutagenesis. Future precision oncology initiatives should investigate the use of targeted treatments early in LFS patients’ disease course.

## Methods

### Whole genome sequencing

Whole genome sequencing was performed on fresh frozen tumor-derived DNA and matched normal (blood-derived or fibroblast cell line) DNA on HiSeqX to an average depth of 40X. In addition to tumor samples sequenced in house, raw sequencing data were downloaded for 8 adrenocortical tumor-normal matched genomes^9^ and 7 osteosarcoma tumor-normal matched genomes^28^ and re-processed from FASTQ using in house pipelines, see below.

### Alignment, variant calling and filtering

#### For driver analysis, primary mutational signature analysis and multiregion phylogenetic reconstructions

FASTQ files were aligned to the hg19 reference genome using BWA-mem (v0.7.8). Duplicates were marked with Sambamba (v0.7.0), and base recalibration and realignment was performed using GATK (v4.1.3) and GATK(V3.8) respectively. Substitution and indel calls were made using MuTect2 from GATK (v4.1.9). Structural variants (deletions, duplications, inversions and translocations) were called using delly (v0.7.1 s)^29^, with a minimum of 4 discordant reads in the tumor required to call each SV. Clonal and subclonal CNVs were called using Battenberg (v3.2.2). All mutation calls (SNVs and SVs) were filtered as previously described using in house pipelines^30^. Briefly, we required a minimum depth of 10X in the tumor and normal with ≤1 reads supporting the variant in the matched normal. We also removed those variants found in a panel of normal non-neoplastic tissue sequenced (*n* = 133) and analysed using the same methods, as well as those that failed at least 2 of 4 cutoffs for non-unique mapping (<70% of reads at locus map uniquely), multi-mapping clusters (seen in tumor and matched normal), excessively high mapping depth (vs the average of the normal chromosome) and those present in low complexity regions (DUST score > 60). Mutation burden was calculated per megabase, the count of all coding and non-coding variants which passed the above QC filters were divided by a genome size of 2800 Mb.

#### For remaining analyses

FASTQ files were aligned to the hg19 reference genome using BWA-mem (v0.78). Duplicates were marked with Picard (v1.1.08), and base recalibration and realignment was performed using GATK (v2.8.1). Merged in silico bulk sequencing BAMs were generated by processing together all WGS FASTQ files from multiple regions to generate a single BAM file for each tumor. BAM files generated from individual tumor regions as well as in silico merged BAMs were processed for variant calling and filtering as described below. Substitution and indel calls were made using MuTect2 from GATK (v3.4.0). Structural variants (deletions, duplications, inversions and translocations) were called using delly^29^ with a minimum of 4 discordant reads in the tumor required to call each SV (v0.7.1). Clonal and subclonal CNVs were called using Battenberg v3.2.2. All mutation calls (SNVs and SVs) were filtered as previously described using in house pipelines^30^. To reiterate, we required a minimum depth of 10X in the tumor and normal with 0 reads supporting the variant in the matched normal. We also removed those variants found in a panel of normal non-neoplastic tissue sequenced (*n* = 133) and analysed using the same methods, as well as those that failed at least 2 of 4 cutoffs for non-unique mapping (<70% of reads at locus map uniquely), multi-mapping clusters (seen in tumor and matched normal), excessively high mapping depth (vs the average of the normal chromosome) and those present in low complexity regions (DUST score > 60). Mutation burden was calculated per megabase, the count of all coding and non-coding variants which passed the above QC filters were divided by a genome size of 2800 Mb.

### Driver mutation identification

SNV and indel calls passing the above filters were analysed using Cancer Genome Interpreter^31^. Variants predicted as Tier1 or Tier2 driver variants in solid tumors as well as those variants annotated as known drivers in solid tumors were classified as driver variants. Filtered structural variants, from delly, with breakpoints in known tumor suppressor genes were also included.

### Telomere analysis

Telseq (v0.0.1), a software package designed to calculate mean telomere length from WGS data, was run on each tumor and matched normal genome^32^. TelSeq estimates mean telomere length by counting the number of reads with telomeric repeats (TTAGGG), divided by GC-adjusted coverage multiplied by the mean chromosome size. We used a read-length of 150 and a threshold of at least 12 telomeric repeats per read. This threshold has previously been found to give the best performance for TelSeq telomere length estimates generated using HiSeqX WGS data (150 bp paired-end reads) in a comparative analysis vs conventional q-PCR telomere length estimation^33^. T/N ratio was calculated by dividing the estimated telomere length (in kb) of the tumor genomes by the matched normal genome.

### Mutational signature analysis

Mutational signature analysis was performed using SigProfiler, a non-negative matrix factorization-based tool to identify mutational signatures de novo from cancer genomic sequencing cohorts^16^. For the primary mutational signature analysis, SigProfiler was run on filtered SNV calls following HotSpot analysis to distinguish clustered and non-clustered mutations. Clustered and non-clustered mutations were analyses separately. Standard parameters for SigProfiler were used with 200 bootstrapping iterations, a minimum number of 2 signatures and a maximum number of 25 signatures. The best number of signatures selected was 7 for unclustered mutations and 3 for clustered mutations, in order to maximize minimum stability and minimize the mean L2 %. These signatures were subsequently decomposed into previously reported SBS signatures and/or novel signatures using SigProfiler^16^.

For the purposes of identifying SBS1 and SBS5 mutations prior to or after whole chromosome 17 copy number gain, mutations were analyzed irrespective of clustering. The best number of signatures selected in this analysis was 8, in order to maximize minimum stability and minimize the mean L2 %. These signatures were subsequently decomposed into previously reported SBS signatures and/or novel signatures using SigProfiler, with 18 SBS signatures identified. C- > T substitutions at CpG sites, as well as all other SNVs with a >50% probability of being contributed by SBS1 or SBS5 according to SigProfiler, were used. DBS signatures were also analyzed using PhyloWGS with standard parameters.

### Mutation timing analysis

Mutation timing was performed using SNV and battenberg copy number data (described above) with MutationTimeR version 1.0 in R.3.6.1 using default parameters (bootstrap of 10)^24^. Mutation time of the first copy number gain occurring that overlapped *TP53* was used for all analyses. PCAWG data was obtained from https://dcc.icgc.org/pcawg.

### Subclonal analysis of individual tumor regions

Filtered SNVs as described above, along with battenberg derived subclonal CNV calls were used as inputs for PhyloWGS in single sample mode^34^. A maximum of 5000 SNVs were used for each sample, with random subsampling when necessary. 10 chains were run in parallel for each sample with a burnin of 1000 iterations followed by 2500 Markov chain Monte Carlo iterations. The best tree was selected as the tree at the point of maximum density when plotting the co-clustering index versus branching degree (branching index/branching index + linearity index). The clonal fraction was determined as the number of mutations in cluster 1 divided by the total number of SNVs.

### Phylogenetic reconstruction of multiple tumor regions

In order to explore and investigate temporal emergence of driver variants in different regions resected from single tumor samples, clonal analysis on single nucleotide variants (SNVs), small insertions and deletions (INDELs) and copy number variations (CNVs) was performed on whole-genome sequenced multi-region tumor samples using PyClone-VI, which clusters mutations based on cellular prevalence estimates using tumor purity of each sample and sex of each patient followed by inferring phylogenetic relations among these clusters using the Pairtree tool^35^. To understand and visualize mutational burden overtime, all mutations were included while calculating mutational burden for each sample and cluster. Sequencing coverage at each locus was sampled from the Poisson distribution and VAF was simulated by sampling the number of mutant reads from a binomial distribution based on the simulated coverage and success rate adjusted by purity and ploidy to give the accurate CCF distributions (clonal and subclonal). To include all samples regardless of tumor purity, no threshold for tumor purity was set during the clonal analysis. Pairtree was not only able to detect all clonal and subclonal clusters at all SNV burden levels, but it also did not assign any of the non-truncal variants to the truncal cluster, especially at this relatively low WGS coverage. This suggests that the non-truncal variants detected in the WGS dataset are not a result of noise in the data.

### LFS dermal fibroblast cell culture and DNA extraction

Skin biopsy samples were received and dissected before being incubated with collagenase in 37 °C incubator for 1.45 h. The samples were then centrifuged at 370 g for 10 min and the supernatant was removed. Trypsin/EDTA were added to the pellets and the cells were pipetted for homogeneity and incubated at 37 °C for 30 min. These cells were then centrifuged and washed followed by incubation with alpha-MEM and 20% FCS. These cells were sub-cultured in 1 week. The cells were incubated at 37 °C in a humidified atmosphere of 5% CO2 and were maintained in DMEM (Biological Industries, Bet-Haemek, Israel) supplemented with 10% FCS and 60 mg/ml penicillin, 100 mg/ml streptomycin. The fibroblast cells were passaged for at least 37–38 passages.

DNA was extracted from cell pellets by Zymo Research g-DNA mini kit as instructed by the manufacturer at early and late passages. Briefly, 50,000 cells from different passages were seeded in 6 cm plates, after the cells became confluent. The cells were then washed, trypsinized and collected in a pellet form. The pellets were then lysed using DNA lysis buffer by vortexing and incubation for 5–10 min. The cell lysates were passed through the Zymo spin column and centrifuged at 10,000 g for 1 min. The cells were washed by g-DNA wash buffer at the same speed and then eluted in 50 µl elution buffer. All steps were taken at room temperature. Finally, DNA was then sequenced and variants called as described above for tumor DNA.

### LFS dermal fibroblast immunofluorescence

LFS fibroblasts were grown on coverslips and fixed with freshly prepared 4 % paraformaldehyde at passage 12 (early), 26 (middle) and 36 (late). Subsequently, the cells were rinsed three times with 1x PBS, washed thrice with 1x PBS with 0.5% Tween 20 and blocked overnight with 5% Bovine Serum Albumin at 4 °C. Blocking was followed by staining with the anti-mutant p53 antibody (ab32049 (Y5), abcam, 1:500 dilution) at RT in a humidified chamber for 1 h. 100ul of diluted solution, which amounts to 200 ng of primary antibody, was used per coverslip.

Incubation with secondary antibody (AF488 Thermo, cat #A35552, 1:200 dilution) was also carried out under similar conditions. 100ul of diluted solution, which is equivalent to 0.5ug of secondary antibody, was used per coverslip. The cells were incubated for 5 min with 1x PBS containing 1 μg/ml DAPI, washed thrice with PBS, and mounted on microscopic slides using mounting media (Fluoromount G, Southern Biotech). The prepared slides were analyzed by using Zeiss LSM800 confocal laser-scanning microscope. At least 150 cells were analyzed for the experiment.

### Deep exome sequencing

Exome sequencing was performed on DNA derived from a 6 mg sample of fresh frozen nonmalignant colonic tissue from the resection surgery for tumor 5524. 4600 ng of DNA was extracted. Exome capture was performed using Agilent SureSelect Human All Exon V5. Following exome capture, DNA was sequenced on 5 NovaSeq lanes to a depth of 5,517x for the nonmalignant colon derived DNA and a depth of 1,426x for the blood-derived DNA. Variants were called and filtered as described above for driver variant detection, with the exception of the depth filter which was required to be within 2 standard deviations of the interval depth.

### ddPCR analysis

Digital droplet PCR analysis was performed on DNA derived from fresh frozen nonmalignant colon tissue and blood-derived DNA. ddPCR analysis was performed using the Bio-Rad QX200 systemn with custom TaqMan probes. Primers and reporter dye probes are listed in Supplementary Data 4 for each variant assessed.

### Consent statement

Written informed consent was provided by all participants or their legal guardians, where appropriate, for this study as well as the publication of anonymized individual data. No compensation was provided to participants. Informed consent to publish individual data that does not include personal health identifiers (including name, date of birth) was freely obtained. All clinical data is anonymized in the manuscript.

### Ethics statement

The study protocol was approved by The Hospital for Sick Children Research Ethics Board and complies with all relevant ethical regulations.

### Reporting summary

Further information on research design is available in the Nature Portfolio Reporting Summary linked to this article.

## Supplementary information

Supplementary Information

Description to Additional Supplementary Information

Supplementary Data 1

Supplementary Data 2

Supplementary Data 3

Supplementary Data 4

Reporting Summary

## Data Availability

The raw WGS and WES data generated in this study have been deposited in the EGA database under controlled access at accession code EGAS00001005982. The St Jude’s adrenocortical tumor sequencing data used in this study are available in the EGA database under controlled access at accession code EGAS00001000257. The previously published osteosarcoma sequencing data is available in the EGA database under controlled access at accession code EGAS00001000196. Access to the raw WGS and WES data described in this paper are available as controlled access as per our institution’s policies meant to prevent study participant identification. Access may be gained through contacting the data access committee listed on the EGA website, which requires submission of a research study proposal demonstrating relevant ethics oversight and a plan for safeguarding of data. Access to the data is available for 1 year once access has been granted. Source data are provided with this paper.

## Source data

Source Data

## Code availability

In-house pipelines used in this study are available at https://github.com/shlienlab.
