# Supplementary material for: Germline TP53 mutations undergo copy number gain years prior to tumor diagnosis
Source: Nat Commun. 2023 Jan 5;14:77. doi: 10.1038/s41467-022-35727-y (PMC9816166; doi:10.1038/s41467-022-35727-y)
Supplement: Supplementary file 1 — Supplementary Information [file 41467_2022_35727_MOESM1_ESM.pdf]

Supplemental Figures and Tables

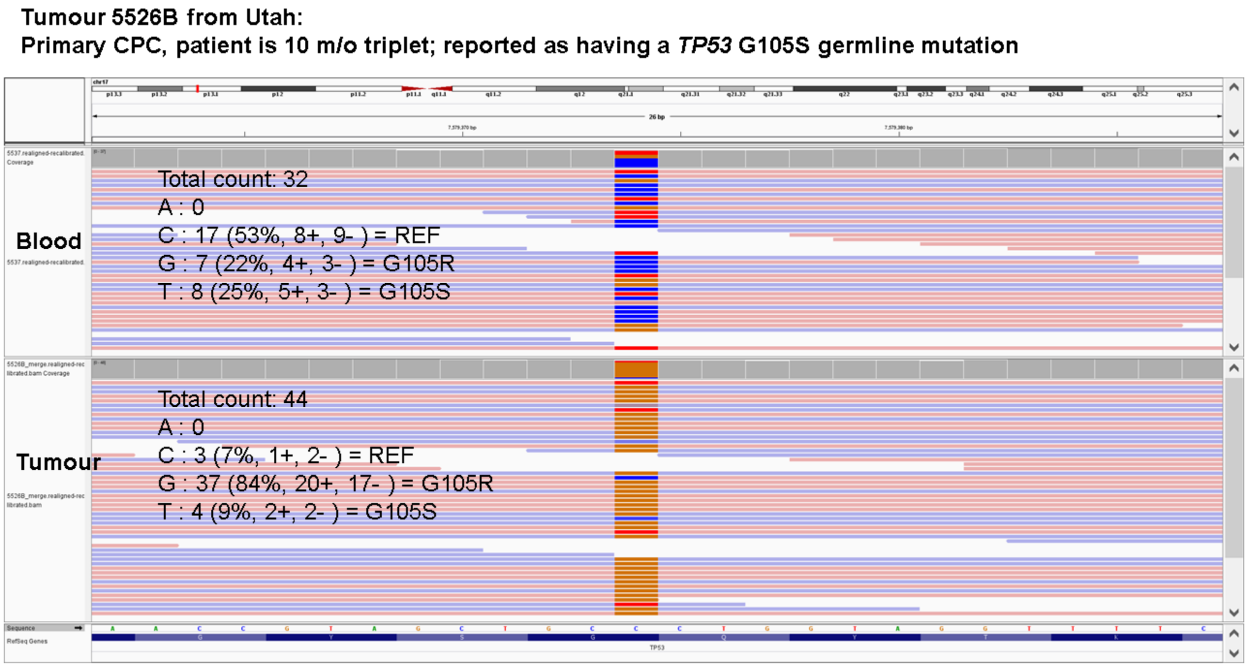

Fig. S1.

Triallelic *TP53* mutation in patient with choroid plexus carcinoma. IGV screenshot of triallelic *TP53* site in blood derived DNA (top) and tumor-derived DNA (bottom). Light red and light blue reads indicate forward and reverse strands, respectively. Red, blue and orange lines show presence of C (reference), T and G alleles respectively at chr17:7,379,374.

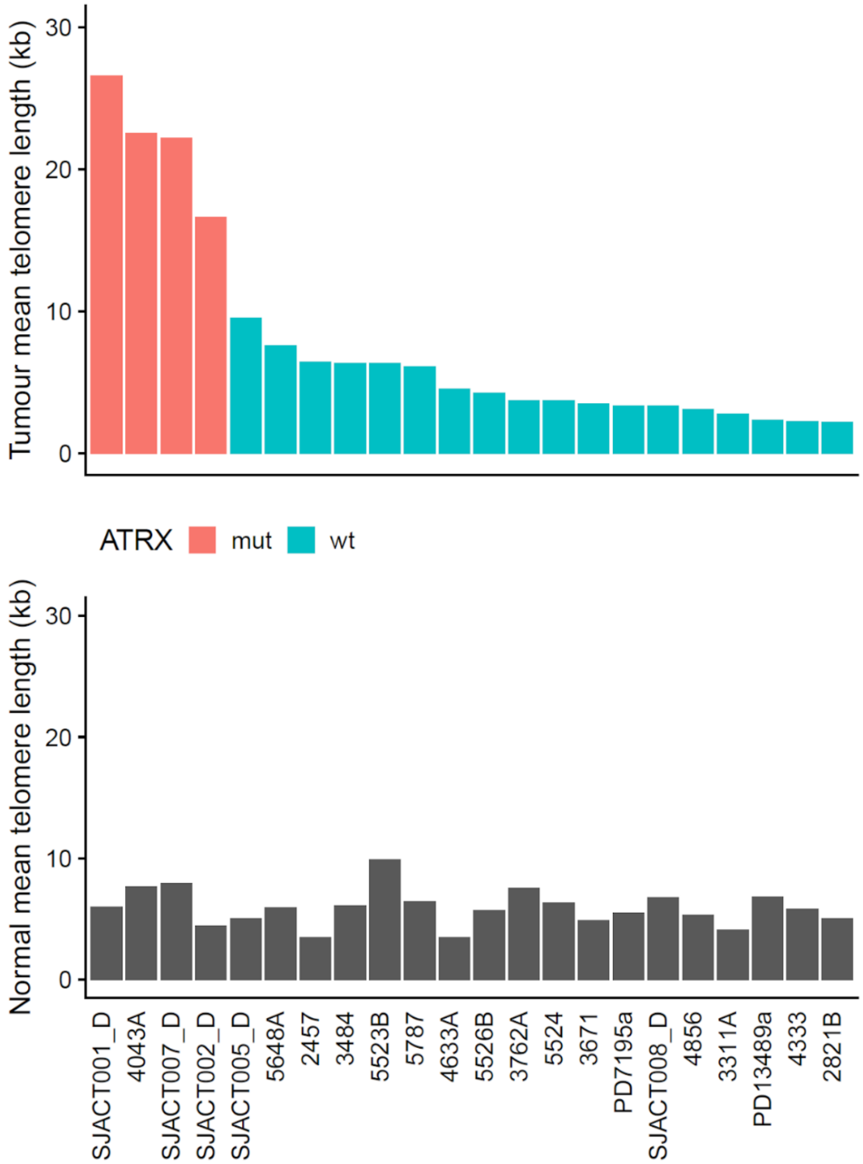

Fig. S2.

Mean telomere length of LFS tumors and matched normal by somatic ATRX mutation status. Tumor (top) and normal (bottom) mean telomere lengths in kb, estimated by whole-genome sequencing. Red bars indicate tumors with *ATRX* mutations. Blue bars indicate tumors wildtype for *ATRX*.

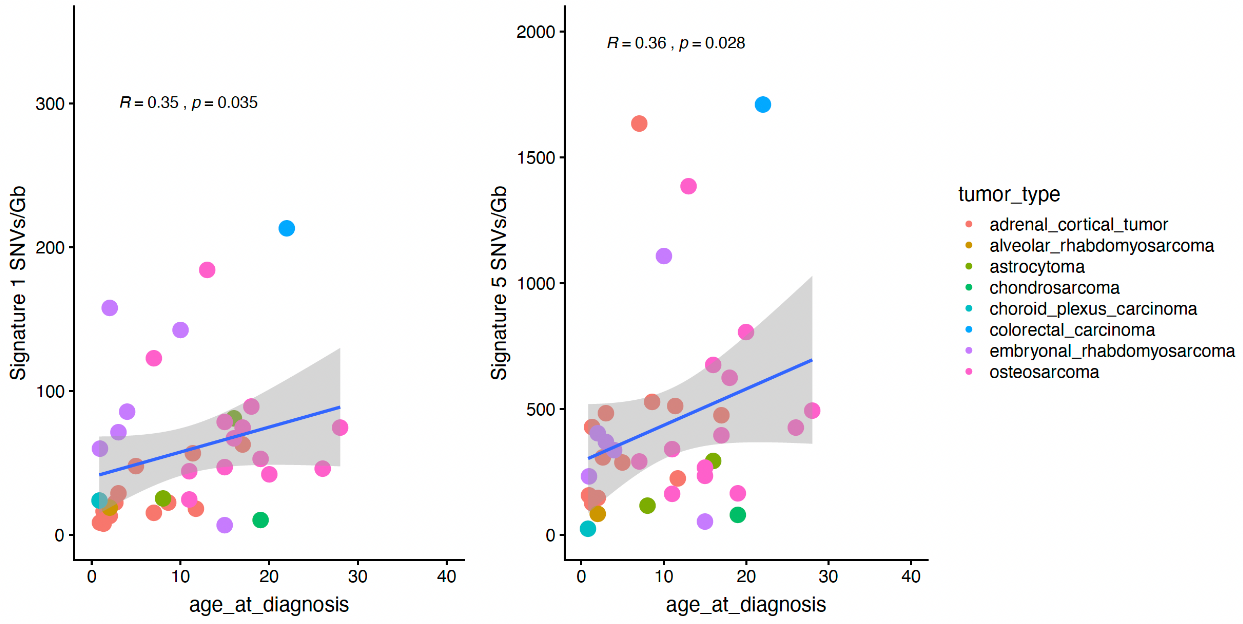

Fig. S3.

Correlation of SBS1 and SBS5 with age in LFS tumor cohort. Scatter plots of tumors age at diagnosis vs the number of SNVs /Mb due to Signature 1 (left) and Signature 5 (right). Colors indicate tumor type. Linear regression lines and Pearson correlation shown.

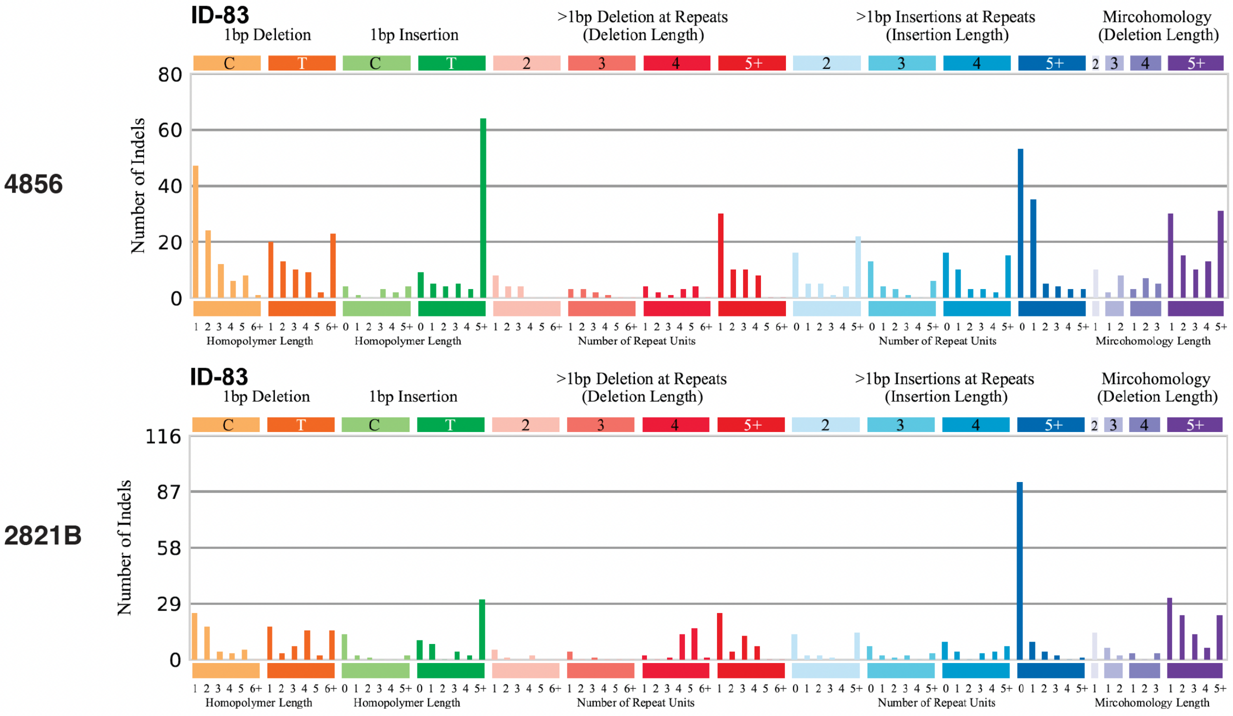

Fig. S4. Microhomology indel presence in tumors with SBS3 BRCA signature. Fig. S10. Bar charts showing 78 classes of indels depicted as in Alexandrov et al., 2020 for tumors 4856 (top) and 2821B (bottom).

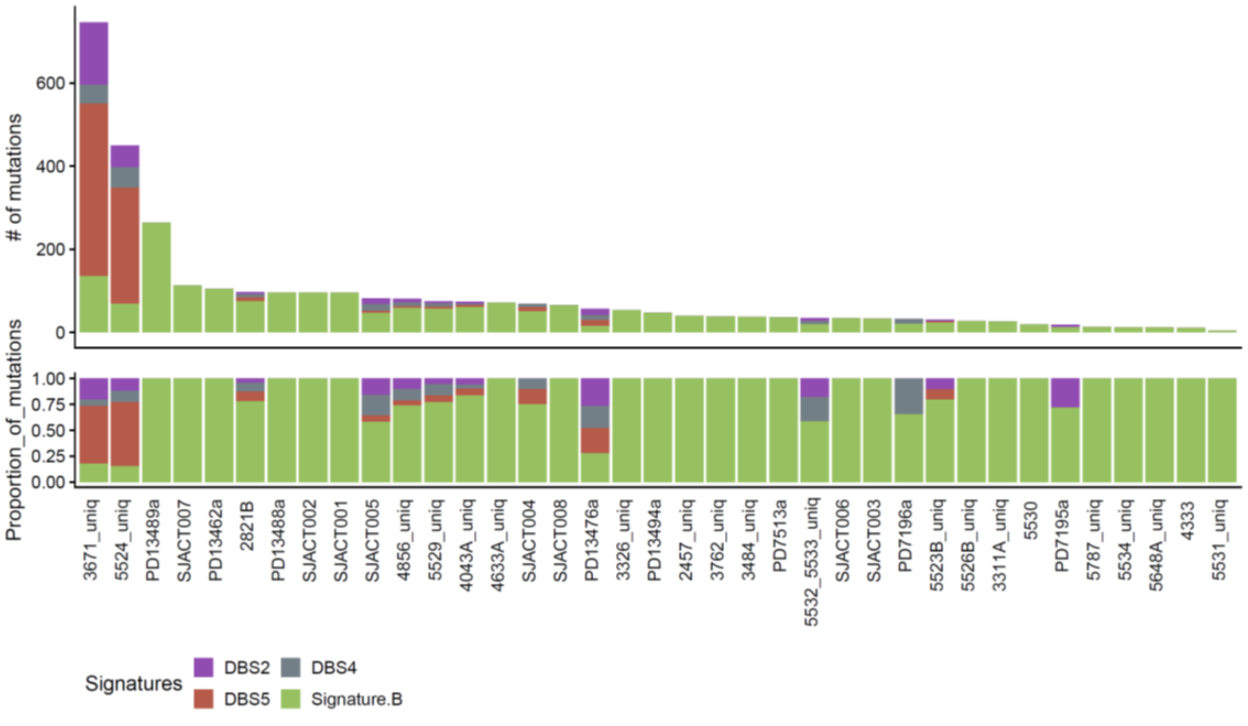

Fig. S5.

High frequency of DBS5 in samples with previous platinum exposure. Dinucleotide base substitution signatures shown by total count (top) and as a proportion (bottom) of all dinucleotide substitutions per tumor. Color of each bar indicate the signatures. DBS2 (purple) has been associated with a variety of exogenous and endogenous mutagens. DBS4 (grey) is a commonly observed signature of unknown aetiology. DBS5 (red) has been associated with platinum-based chemotherapy treatment. Signature B corresponds to an uncharacterized signature.

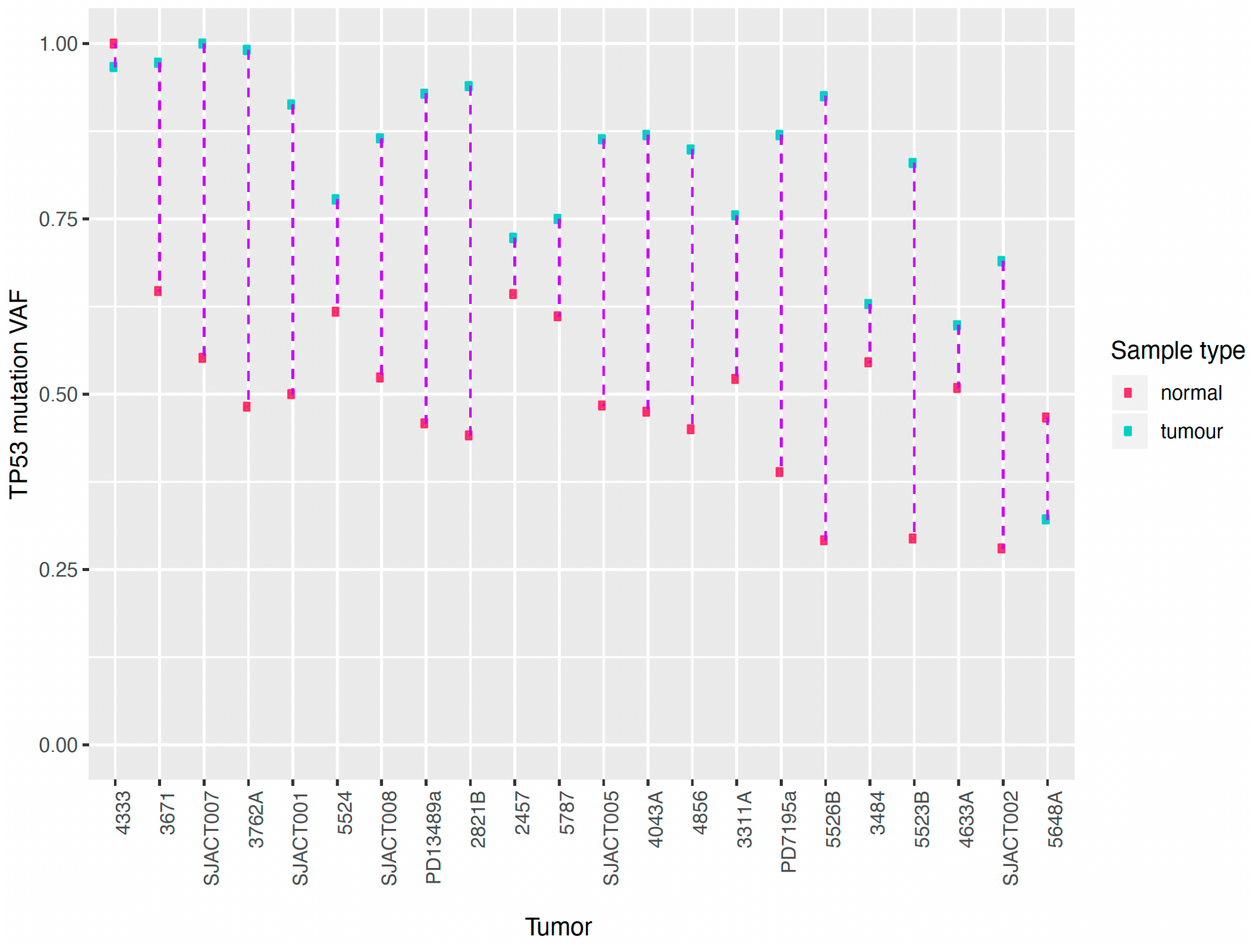

Fig. S6.

Variant allele fraction of *TP53* mutation in tumor and matched normal. Tumors plotted by variant allele fraction of *TP53* mutation from whole genome sequencing data generated in normal (red) and tumor (blue).

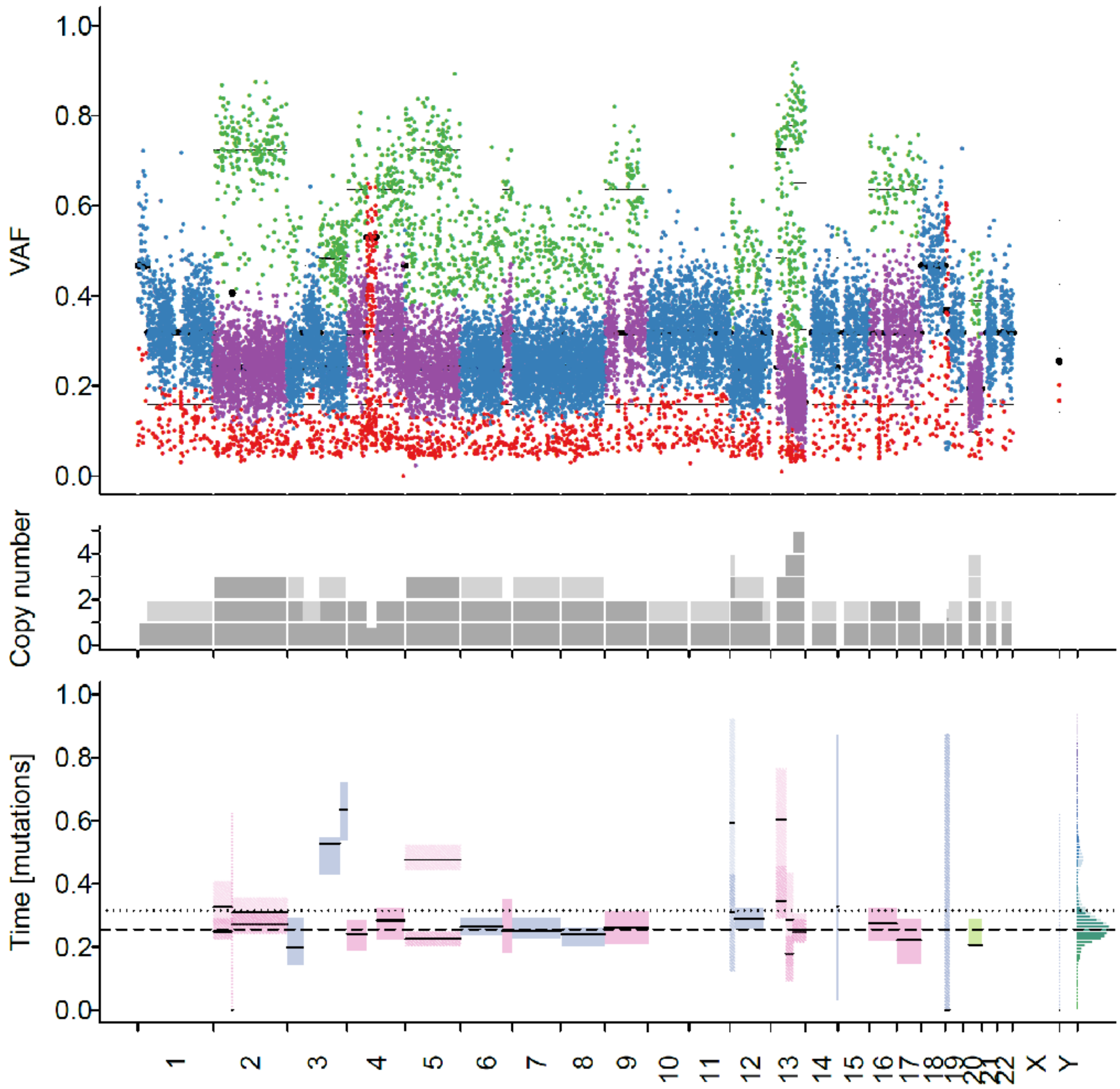

Fig. S7.

Example of mutationTimeR results for an LFS tumor. Top: All SNVs plotted by VAF. Colors indicate clonality of mutations (green = clonal early, blue = clonal unspecified, purple = clonal late, red = subclonal). Middle: Major (dark grey) and minor (light grey) copy number plot. Bottom: Mutational timing of regions undergoing copy number gain. Mutational time on y-axis corresponds to fraction of somatic SNVs occurring before the copy number gain. Colored bars are copy number gain, with shading indicating the 95% confidence interval.

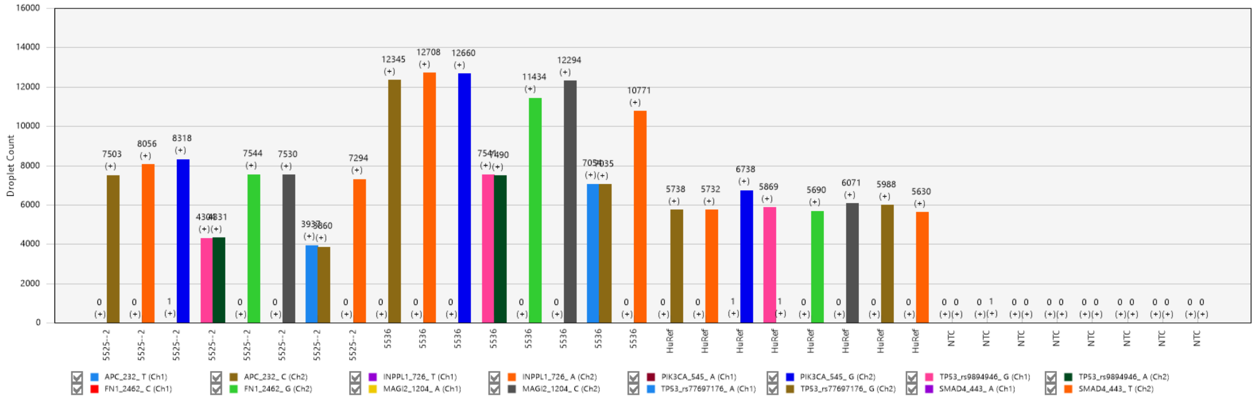

Fig. S8.

ddPCR analysis of heterozygous SNPs and driver mutations identified in tumor 5524, in normal colonic tissue from same patient. Bar chart showing droplet counts of mutant and wildtype alleles, colored by allele. Note *TP53* variants in this analysis were germline heterozygous SNPs.

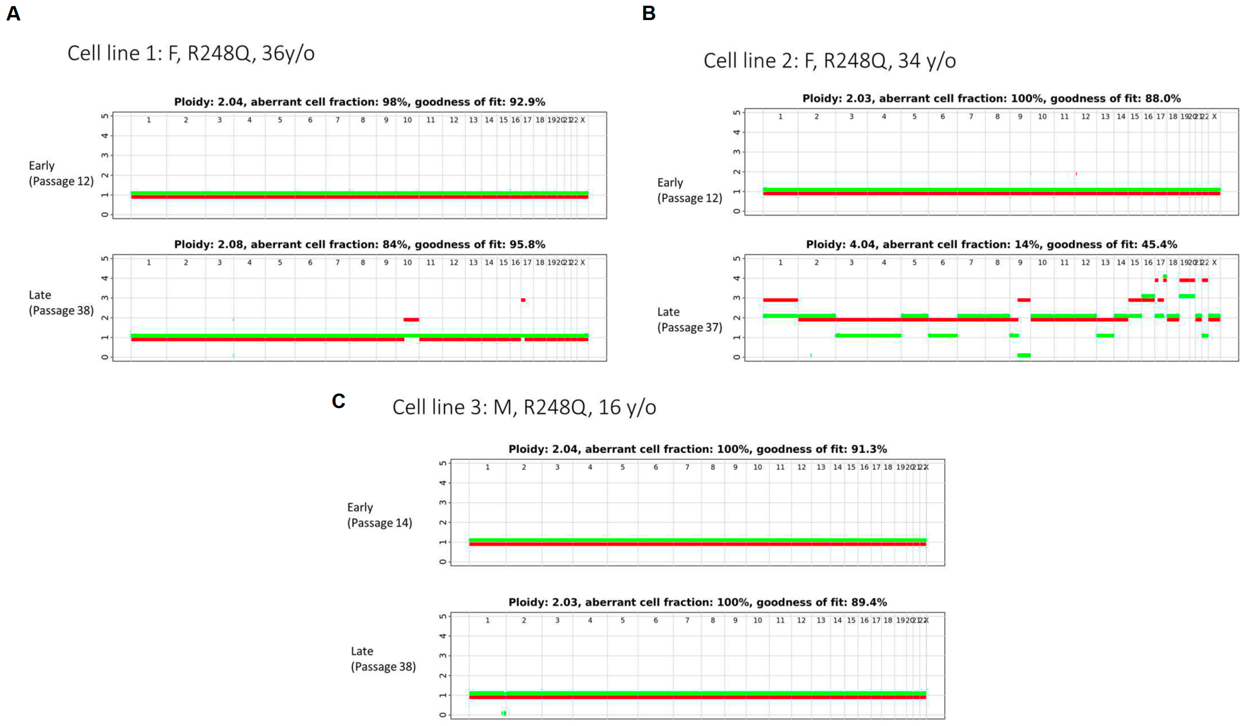

Fig. S9.

Spontaneous copy number gain of LFS fibroblast cell lines. Red and green lines denote copy number of major and minor alleles, respectively. A) Cell line 1 with copy number gains of 17p and 10q. B) Cell line 2 with genome duplication and many additional copy number changes including gain of 17p. C) Cell line 3 exhibits diploid genome.

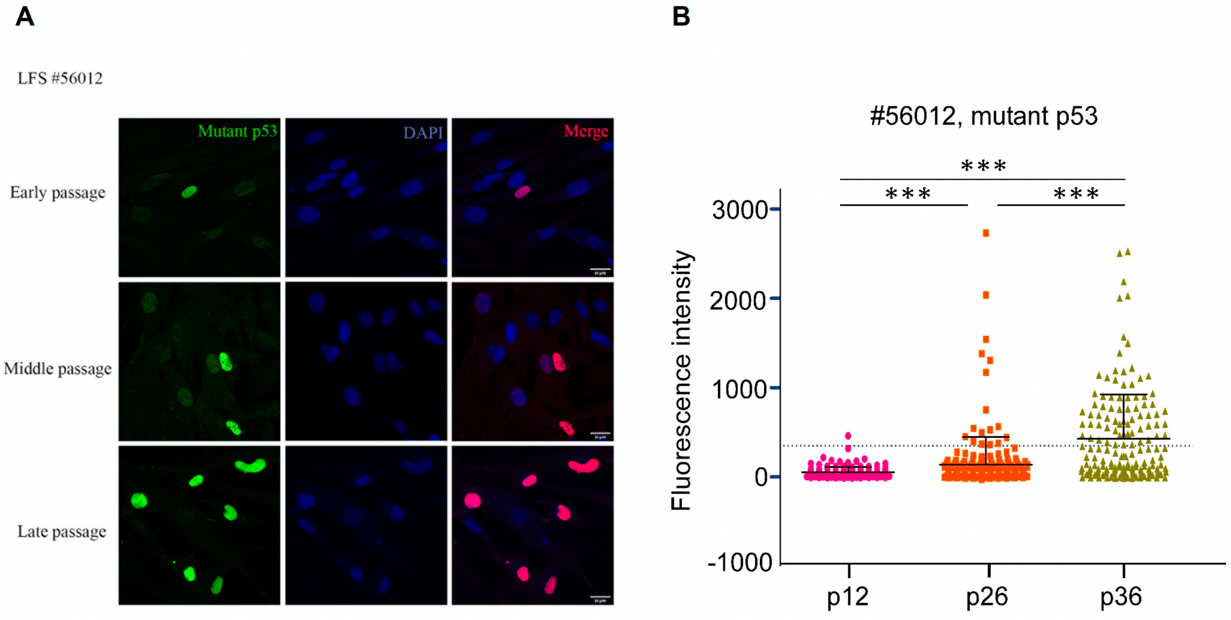

Fig. S10.

A small fraction of cells show accumulation of mutant p53 in earlier passages evidently depicting WT p53 LOH. A) LFS fibroblasts #56012 were asynchronously grown till passages 12 (early), 26 (middle) and 36 (late), then fixed and stained with mutant p53 antibody. The stained cells were visualized and imaged by confocal microscopy. Nucleus was stained with DAPI. Scale, 20 µm. Three independent experiments were conducted with similar results. **B)** Quantitation of the mutant p53 staining was done using Image J software ($p<0.0001$, $n>150$). The data are presented as the average ± standard deviation of three independent experiments.

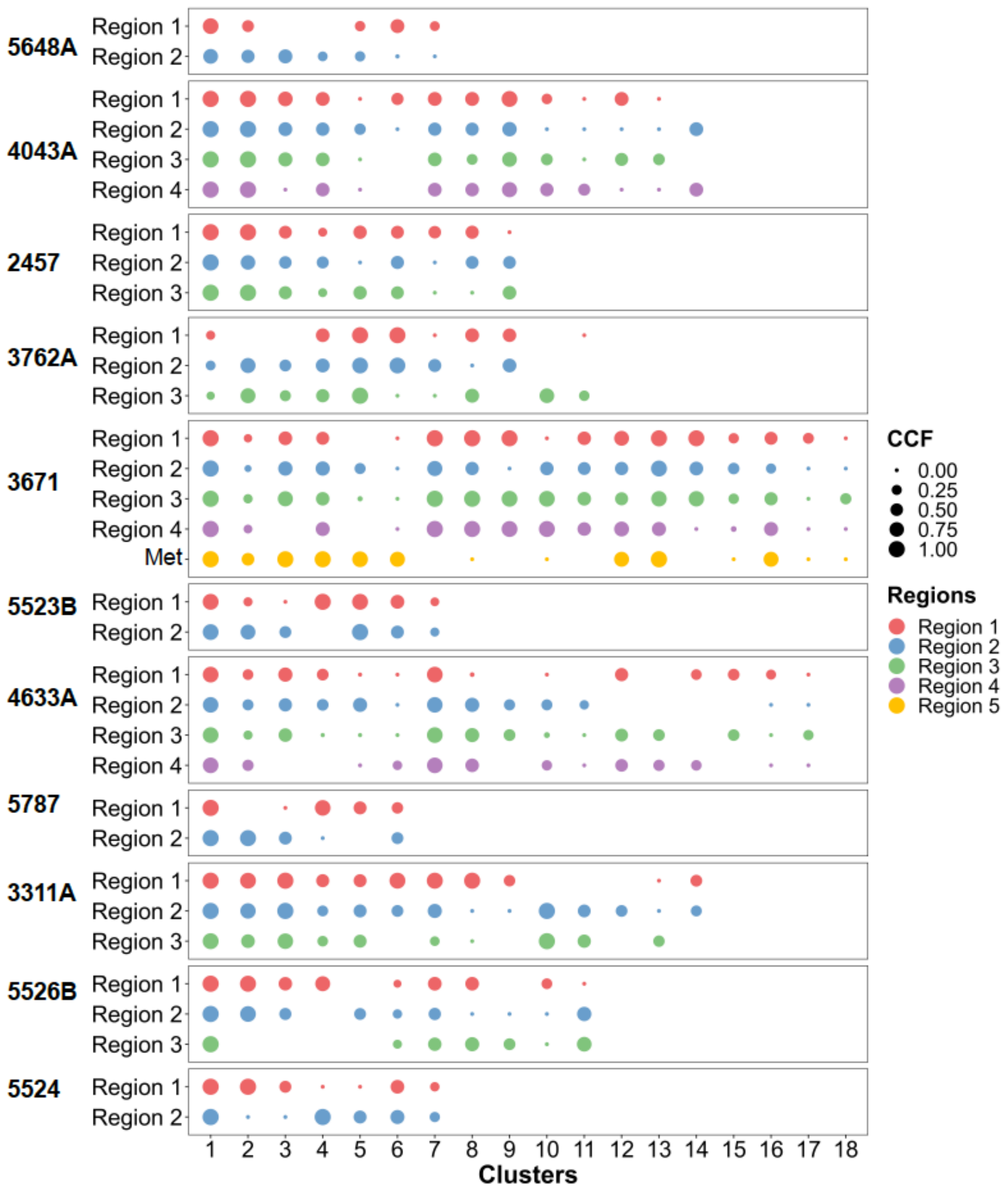

Fig. S11.

TMB of subclonal clusters derived from LFS tumors multiregion WGS phylogenetic reconstruction. Circle plots, with subclonal cluster number on X axis and tumor region on Y axis. Circles are colored by tumor region. Circle diameter corresponds to TMB for each cluster for each tumor region.

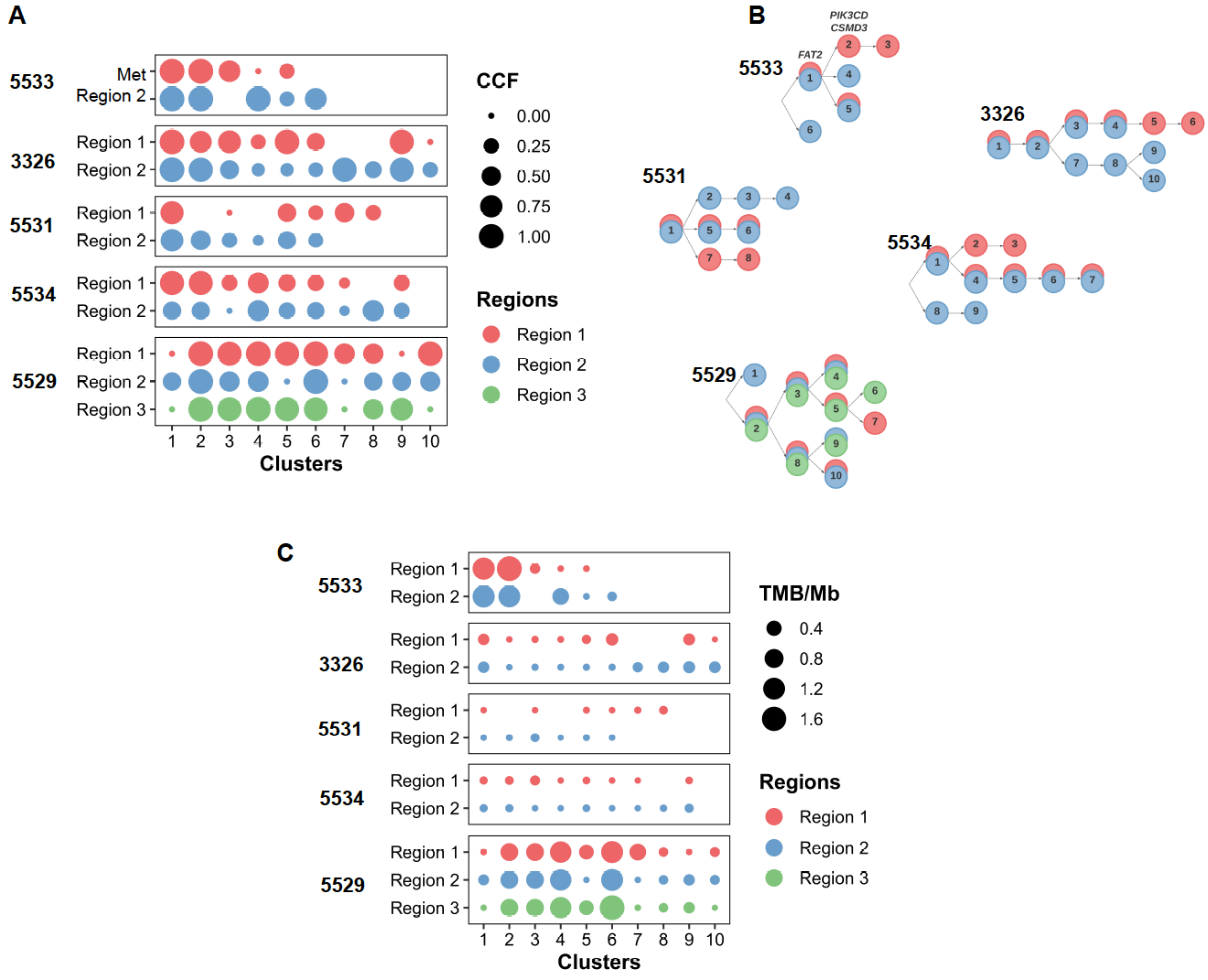

Fig. S12.

Phylogenetic reconstruction of non-LFS tumors using multiregion WGS. A) Circle plots, with subclonal cluster number on X axis and tumor region on Y axis. Circles are colored by tumor region. Circle diameter corresponds to cancer cell fraction for each cluster for each tumor region. B) Phylogenetic tree reconstructions of each multiregion sequenced tumor with colors corresponding to tumor region, numbers corresponding to cluster number and annotated with location of driver mutations. C) Circle plots, with subclonal cluster number on X axis and tumor region on Y axis. Circles are colored by tumor region. Circle diameter corresponds to TMB for each cluster for each tumor region.

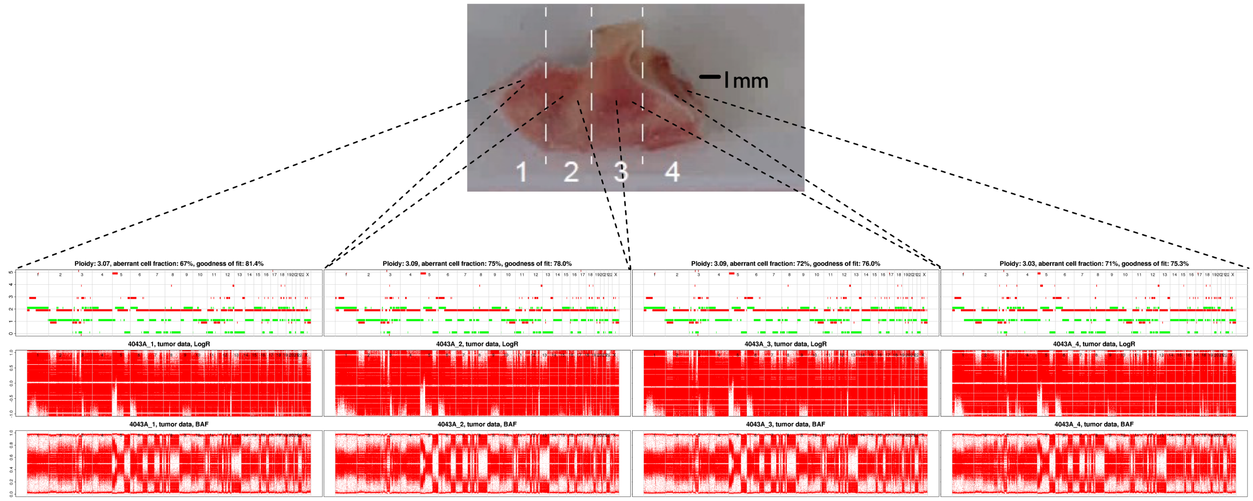

Fig. S13.

Identical pattern of chromothripsis detected in 4 regions of tumor 4043A. (Top) Gross pathology image of tumor 4043A (osteosarcoma), with white dashed lines indicating how specimen was dissected into four sections. (Below) Battenberg copy number plots indicating nearly identical copy number patterns across 4 regions. Red and green lines represent major and minor copy number alleles, respectively. Middle plots indicate LogR ratio of SNPs in each sample. Bottom plots indicate beta allele frequency (BAF) of individual SNPs in each sample.

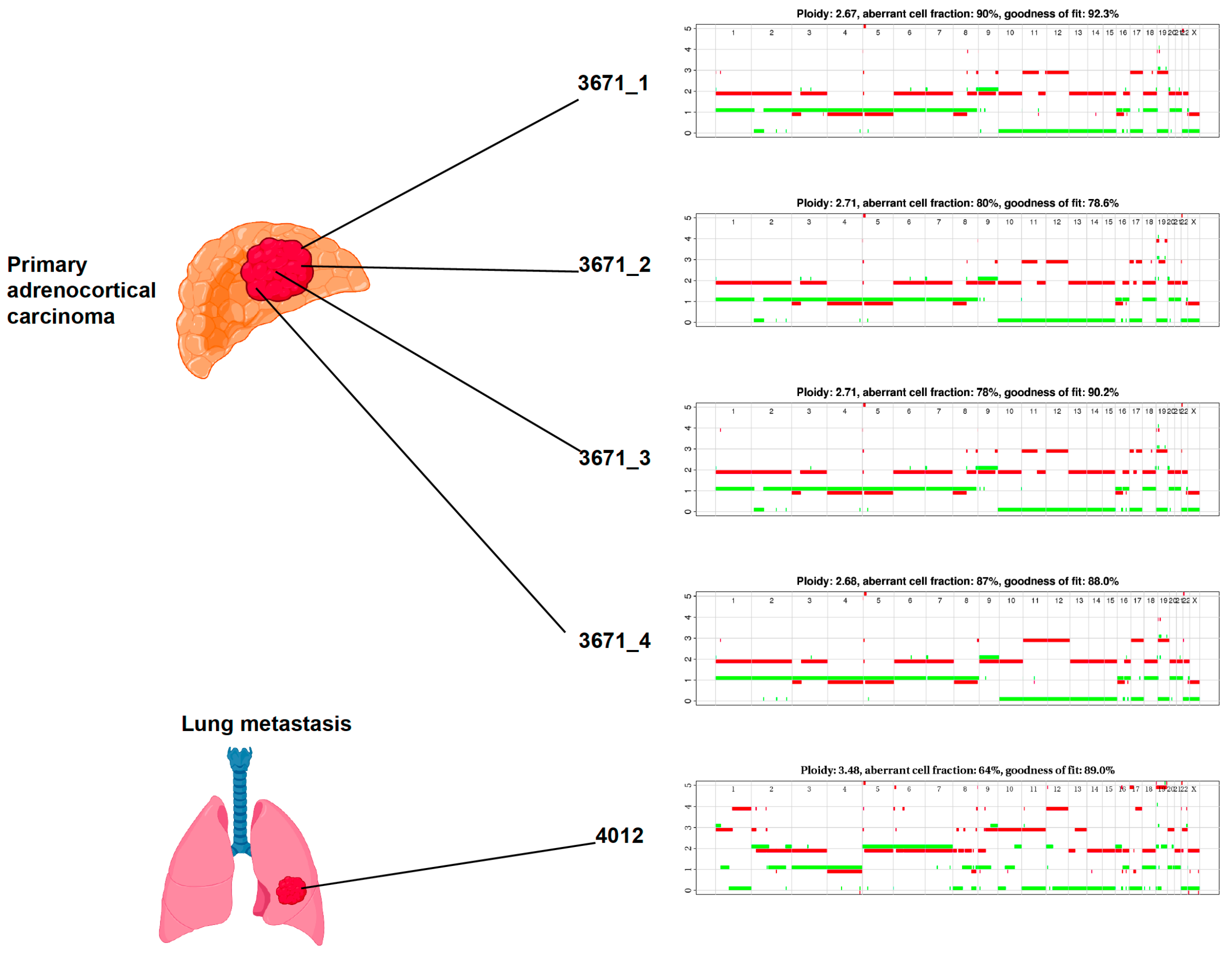

Fig. S14. Copy number variant comparison of primary and metastatic adrenocortical carcinoma in LFS patient

Copy number plots derived from WGS for 4 tumor regions of primary adrenocortical carcinoma, 3671, as well as a lung metastasis, 4012. Red and green lines represent major and minor copy number alleles, respectively.
