# Supplementary material for: Germline TP53 mutations undergo copy number gain years prior to tumor diagnosis
Source: Nat Commun. 2023 Jan 5;14:77. doi: 10.1038/s41467-022-35727-y (PMC9816166; doi:10.1038/s41467-022-35727-y)
Supplement: Supplementary file 2 — Description to Additional Supplementary Information [file 41467_2022_35727_MOESM2_ESM.pdf]

## Description of Additional Supplementary Files

**Supplementary Data 1**

Anonymized clinical information for tumors analyzed in this study, displaying age, sex, TP53 mutation status, tumor type, estimated tumor purity, and regions analyzed.

**Supplementary Data 2**

Somatic SNV and indel driver mutations identified in LFS tumors, displaying tumor ID, chromosomal location and DNA, involved gene, SNV/indel type and corresponding protein change

**Supplementary Data 3**

Somatic SV driver mutations identified in LFS tumors, displaying tumor ID, SV type, chromosomal locations of breakpoints and genes involved at breakpoints.

**Supplementary Data 4**

Custom primers and reporter dye probes used in ddPCR analysis of nonmalignant colon tissue and blood-derived DNA
