# Supplementary material for: Germline TP53 mutations undergo copy number gain years prior to tumor diagnosis
Source: Nat Commun. 2023 Jan 5;14:77. doi: 10.1038/s41467-022-35727-y (PMC9816166; doi:10.1038/s41467-022-35727-y)
Supplement: Supplementary file 7 — Reporting Summary [file 41467_2022_35727_MOESM7_ESM.pdf]

nature portfolio

Corresponding author(s): Adam Shlien & David Malkin

Last updated by author(s): August 16, 2022

Reporting Summary

Nature Portfolio wishes to improve the reproducibility of the work that we publish. This form provides structure for consistency and transparency in reporting. For further information on Nature Portfolio policies, see our Editorial Policies and the Editorial Policy Checklist.

Statistics

For all statistical analyses, confirm that the following items are present in the figure legend, table legend, main text, or Methods section.

n/a	Confirmed	
☐	☒	The exact sample size (*n*) for each experimental group/condition, given as a discrete number and unit of measurement
☐	☒	A statement on whether measurements were taken from distinct samples or whether the same sample was measured repeatedly
☐	☒	The statistical test(s) used AND whether they are one- or two-sided<br>*Only common tests should be described solely by name; describe more complex techniques in the Methods section.*
☐	☒	A description of all covariates tested
☒	☐	A description of any assumptions or corrections, such as tests of normality and adjustment for multiple comparisons
☐	☒	A full description of the statistical parameters including central tendency (e.g. means) or other basic estimates (e.g. regression coefficient) AND variation (e.g. standard deviation) or associated estimates of uncertainty (e.g. confidence intervals)
☐	☒	For null hypothesis testing, the test statistic (e.g. *F*, *t*, *r*) with confidence intervals, effect sizes, degrees of freedom and *P* value noted<br>*Give P values as exact values whenever suitable.*
☒	☐	For Bayesian analysis, information on the choice of priors and Markov chain Monte Carlo settings
☒	☐	For hierarchical and complex designs, identification of the appropriate level for tests and full reporting of outcomes
☐	☒	Estimates of effect sizes (e.g. Cohen's *d*, Pearson's *r*), indicating how they were calculated

Our web collection on statistics for biologists contains articles on many of the points above.

Software and code

Policy information about availability of computer code

Data collection	No code was used to collect data for this study.
Data analysis	BWA-mem (v0.7.8), Sambamba (v0.7.0), GATK (v4.1.3), GATK(V3.8), MuTect2 from GATK (v4.1.9), delly (v0.7.1), Battenberg (v3.2.2), Picard (v1.1.08), GATK (v2.8.1), Cancer Genome Interpreter (Accessed on December 13, 2021), Telseq (v0.0.1), SigProfiler (v1.14), MutationTimeR (v1.0), R (v3.6.1), PyClone-VI, PairTree, ggplot2, plyR, dplyR, deconstructSigs, reshape2<br><br>In-house pipelines used in this study are available at https://github.com/shlienlab

For manuscripts utilizing custom algorithms or software that are central to the research but not yet described in published literature, software must be made available to editors and reviewers. We strongly encourage code deposition in a community repository (e.g. GitHub). See the Nature Portfolio guidelines for submitting code & software for further information.

Data

Policy information about availability of data

All manuscripts must include a data availability statement. This statement should provide the following information, where applicable:

- Accession codes, unique identifiers, or web links for publicly available datasets
- A description of any restrictions on data availability
- For clinical datasets or third party data, please ensure that the statement adheres to our policy

The raw sequencing data generated in this study have been deposited in the EGA database under accession code EGAS00001005982 (https://ega-archive.org/studies/EGAS00001005982). The raw sequencing data are available under restricted access to protect the privacy of study participants, access can be obtained by submitting a research application to the data access committee. The processed data generated in this study are provided in the Source Data files. The third party

March 2021

raw sequencing data used in this study are available in the EGA database under accession codes EGAS00001000257 (https://ega-archive.org/datasets/EGAS00001000257), and EGAS00001000196 (https://ega-archive.org/studies/EGAS00001000196).

Field-specific reporting

Please select the one below that is the best fit for your research. If you are not sure, read the appropriate sections before making your selection.

☒ Life sciences ☐ Behavioural & social sciences ☐ Ecological, evolutionary & environmental sciences

For a reference copy of the document with all sections, see nature.com/documents/nr-reporting-summary-flat.pdf

Life sciences study design

All studies must disclose on these points even when the disclosure is negative.

Sample size	The sample size of Li-Fraumeni syndrome patient tumors was not predetermined, but rather the maximum number of available fresh frozen tumor samples available for whole genome sequencing.
Data exclusions	No data were excluded from the analysis.
Replication	Replication was not possible due to the nature of the study and limited number of fresh frozen tumor samples from Li-Fraumeni syndrome patients
Randomization	Randomization was not performed in this study as this is a descriptive study which sought to characterize a rare set of tumors, without a priori hypotheses. The effects of covariates such as age and tumor type were directly assessed in the study, where relevant.
Blinding	Blinding was not performed in this study, as again there was no a priori hypotheses we sought to test in this study. The analyses pipelines did not rely on our subjective interpretation.

Reporting for specific materials, systems and methods

We require information from authors about some types of materials, experimental systems and methods used in many studies. Here, indicate whether each material, system or method listed is relevant to your study. If you are not sure if a list item applies to your research, read the appropriate section before selecting a response.

Materials & experimental systems

n/a	Involved in the study	
☐	☒	Antibodies
☒	☐	Eukaryotic cell lines
☒	☐	Palaeontology and archaeology
☒	☐	Animals and other organisms
☐	☒	Human research participants
☒	☐	Clinical data
☒	☐	Dual use research of concern

Methods

n/a	Involved in the study	
☒	☐	ChIP-seq
☒	☐	Flow cytometry
☒	☐	MRI-based neuroimaging

Antibodies

Antibodies used	Anti-mutant p53 antibody (ab32049 (Y5), abcam)
Validation	ab32049 recognises human mutant forms of p53 but not human p53 wild type. ab32049 showed a negative signal on wildtype p53 cell lines (HepG2, A549, MCF-7) and a positive signal on mutant p53 cell lines (T47-D, Raji, A431) in WB. [From abcam website: https://www.abcam.com/mutant-p53-antibody-y5-ab32049.html]

Human research participants

Policy information about studies involving human research participants

Population characteristics	The population of interest were tumors derived from pediatric and young adult patients (ages 0-30), with and without germline pathogenic variant in TP53, both male and female, from a range of solid tumor types including adrenocortical tumors, rhabdomyosarcomas, osteosarcomas, and gliomas.
Recruitment	Li-Fraumeni syndrome patients were recruited for this study based a known germline pathogenic variant identified in the TP53 gene. For the purposes of this study they must also have had a resected tumor sample which had been preserved fresh frozen. The majority of tumor samples were obtained from tumor banks at large academic centres, in North America,

especially The Hospital for Sick Children in Toronto, Canada and the Huntsman Cancer Centre in Salt Lake City, Utah, which may not represent the global population of patients with Li-Fraumeni syndrome.

Ethics oversight

The Hospital for Sick Children Research Ethics Board provided ethical oversight.

Note that full information on the approval of the study protocol must also be provided in the manuscript.
